# Hypnotism and Hysteria

**Published:** 1892-08-27

**Authors:** 


### THE DOCTOR'S ARMCHAIR.

### HYPNOTISM AND HYSTERIA.

The medical profession in this country has become a little tired of hypnotism. The intellect of the therapeutic John Bull is of the severely practical order, and has all the impatience of its kind. Not so the profession in France. There is nothing your Frenchman loves better than intellectual gladiatorship; and if such gladiatorship is far removed from the sobering control of the concrete, so much the more will it be agreeable to the combatants. Paris and Nancy have long been arrayed against each other on the subject of hypnotism, and their interest in the conflict shows no sign of waning. Rather the opposite; for the French doctors, having probably worn out the patience of the medical editors of their own country, are now invading the professional journals of the great American continent and there carrying on the war.

M. J. Babinski, Chef de Clinique at the Salpêtrière, Paris, has an article in the July number of the *Journal of Nervous and Mental Disease*, which is translated by Dr. W. G. Herdman, Professor of Diseases of the Nervous System in the University of Michigan. Perhaps we may be permitted to say that the mistakes in this article—printer's, editor's, or translator's—are exceedingly numerous; and not only very disfiguring, but to medical men of limited education will be found positively misleading. But this by the way. M. Babinski, it is hardly necessary to say, sees with M. Charcot's eyes. M. Charcot's views on hypnotism are those which most commend themselves to the judgment of straight thinking and practical Englishmen. M. Babinski considers that hypnotism and hysteria are so nearly related as to be almost, if not entirely, twin sisters. He goes so far as to say that it is "impossible not to see a close relation between hypnotism and hysteria, and one would almost have the right to affirm that hypnotism is a manifestation of hysteria." At the other end of the scale is M. Bernheim, who, as a kind of *alter ego* of Dr. Liebéault, represents the Nancy school of hypnotisers. M. Bernheim denies any relationship whatever between hypnotism and hysteria.

This is a pretty, and, on the whole, a fortunate quarrel. It may result in the proper threshing out of the whole subject, a proceeding much to be desired. All sorts of quackery and knavery are beginning to be associated with hypnotism, both within and without the medical profession, and it is eminently necessary that some authoritative pronouncement be made by competent persons who have worked out the subject to its utmost depths.

The first thing to be done in a discussion of this kind is to define the terms. What, on the one hand, is the term "hysteria" to signify? And what, on the other, is "hypnotism" to be held exactly to mean? Of course if the object of the combatants is merely to carry on a wordy wrangle, there is no particular necessity to be precise in the use of terms. But if it be intended to prosecute a real scientific discussion with the view of arriving at truth, then the self-respect of both parties demands that "terms" be precisely defined, and that the definitions agreed upon be strictly adhered to. M. Bernheim shows that he is aware of the danger that may lurk in a definition. "Hysteria" he carefully avoids defining altogether; and "hypnotism" he defines with such delightful vagueness that he can be no more pinned down to an actual meaning than can a butterfly high in the air be secured to a lepidopteral museum card. This, we submit, is not science, but something that should be called by a much less respectable name. It may give a little amusement, even to our soberest readers, if we furnish them with M. Bernheim's definition. "Hypnotism," says this scientific person, "is a certain psychic condition susceptible of being provoked, which brings suggestibility into activity, or exalts it in different degrees; that is to say, the aptitude to be influenced by an idea accepted by the brain, and to realise that idea." This is exquisite! A "certain psychic condition"! Exactly. But we want the "psychic condition" itself defined before we go a single step further. For the real meaning of the definition, if its language is to be interpreted literally, is this and nothing else, that every man, woman, and child ever born into the world is, and always must be, in a hypnotic condition. For every living person, when broad awake, is in a "certain psychic condition"; that condition is "susceptible of being provoked," it brings "suggestibility into activity," and it has "the aptitude to be influenced by an idea accepted by the brain, and to realise that idea."

If we were not determined at all risks to avoid rudeness, we should say bluntly and plainly that M. Bernheim has the most hazy idea of what he is talking about. He uses the word "psychic" like a medical student who has never studied philosophy. Of the actual thing which he intended to define he has the vaguest of all conceptions. How can a man define that which he has never taken the trouble to understand, that which escapes his vision at a thousand different points? If he does not understand a thing, let him have the candour to say so. He may then be scientific in temper, even though he be ignorant of facts. But when a man gives us a cloud of words, and asks us to accept them as the equivalent of things, a small part of which only he has even so much as seen, we do not call him "scientific," but something very decidedly otherwise.

M. Babinski gives reasons, both clinical and chemical or his conclusion that hypnotism and hysteria are very ntimately allied. He tells us, what we already knew,, that, "according to the doctrines at Salpêtrière, hypnotism can be of little service as a curative agent except in hysteria. One can affirm, in any case," he continues, "that most of the troubles cured by this method arise from neurosis. This should be recognised, but it must be acknowledged also that, even in affections of this kind, hypnotic practice does not always give brilliant results." M. Babinski, himself a hypnotiser, be it remembered, compares hypnotic medication to the venerable practice of giving hypochondriacs and other nervous persons potions of distilled water and bread pills, and he considers that in many cases the results will be equally good, whether hypnotism or "bread pills" be employed. The intention in each case is to "suggest" that a cure is being made, and the "suggestion" in each case actually makes the cure. It is pretty plain that in Paris, at any rate, the days of mysterious and miracle-working hypnotism are now numbered.

